# Green and Blue Space Availability and Self-Rated Health among Seniors in China: Evidence from a National Survey

**DOI:** 10.3390/ijerph18020545

**Published:** 2021-01-11

**Authors:** Chensong Lin, Longfeng Wu

**Affiliations:** 1 School of Landscape Architecture, Beijing Forestry University, Beijing 100083, China; linchensong@bjfu.edu.cn; 2 Graduate School of Design, Harvard University, Cambridge, MA 02138, USA

**Keywords:** green space, blue space, self-rated health, senior population, urban and rural areas, China Social Survey

## Abstract

Many empirical studies have shown evidence of multiple health benefits provided by green and blue spaces. Despite the importance of these spaces, investigations are scarce in details for blue spaces rather than green. Moreover, most research has focused on developed regions. A limited number of studies on blue spaces can be found in China with a focus on the city level. Outcomes have been mixed due to varying research scales, methodologies, and definitions. This study relies on a national-level social survey to explore how the self-rated health (SRH) of senior individuals is associated with local green and blue space availability in urban and rural areas. Results indicate that the coverage ratio of overall green spaces and waterbodies around a resident’s home have marginal effects on SRH status in both urban and rural areas. In urban areas, living close to a park can is marginally beneficial for older people’s health. Regarding different types of blue spaces, the presence of a major river (within 0.3–0.5 km) or coastline (within 1 km and 1–5 km) in the vicinity of home negatively affects SRH among the elderly in urban areas. Close proximity to lakes and other types of waterbodies with a water surface larger than 6.25 ha did not significantly influence SRH. These findings not only evaluate general health impacts of green/blue space development on senior populations across the county but inform decision makers concerning the health-promoting qualities and features of different green/blue spaces to better accommodate an aging population in the era of urbanization.

## 1. Introduction

Green and blue spaces are critical components of humans’ living environment. Green space, in the form of parks, grasslands, forests, farmlands, and even backyard gardens, together with blue space, including lakes, rivers, and coasts, can promote people’s well-being in multiple ways. According to a number of literature reviews, there are several potential pathways for how different types of green/blue spaces benefit well-being [1,2,3]. General vegetation, including trees, grasses, bushes, and crops in both natural and artificial green spaces can reduce harmful environmental factors such as air pollution, heat, and noise in the vicinity, and thus improve people’s general health [1,4,5,6,7,8,9,10]. Accessible green spaces, such as parks, with designed squares and amenities, provide vegetated outdoor spaces for physical activities and social gatherings, through which people can promote their physical and psychological wellness [1,2,3,11,12,13,14,15,16,17].

Compared with the widely acknowledged health-promoting benefits of green spaces, studies concerning blue spaces are growing, yet remain scarce. Despite the recognition of the effects of blue space on people’s health, research on blue spaces has revealed largely mixed results due to varying scales, health indicators, definitions of blue space, and approaches to the measurement of blue spaces [18,19,20]. Similar to green space, the presence of blue space can also serve as an environmental harm reduction. All substantial bodies of static or dynamic surface water are confirmed to minimize urban heat stresses [2,5]. The waterbodies’ capability of reducing air pollution is complex [2]. Studies have found that sea breezes might disperse pollutants in the air [21], while such outcomes may be highly dependent on wind direction, strength, and existence of pollution sources in the sea (e.g., aerosolized toxins from harmful algal blooms) [22]. Easily accessible waterbodies such as riverfronts or coastal/lake shorelines with well-designed/maintained walkable trails can encourage physical activities such as walking, jogging and even water surface sports and be popular venues for social gatherings [2,23,24], and thus tend to benefit people’s health [25]. However, their health-promoting effects are largely mixed. While studies in England, France, and Australia support the higher likelihood of walking and other water sports for people living close to rivers, oceans or lakes [24,26,27,28], observations in the US and China found no differences on physical activities between those living close to or further away from waterbodies [29,30]. Such inconsistency might be associated with features (e.g., lacking accessibility) and qualities (e.g., water pollution) of blue spaces as well as personal socioeconomic factors and physical constraints [2,3,19]. Blue spaces are also known to reduce stress and restore depleted cognitive abilities, which is bolstered by both laboratory and field experiments [1,2]. For example, in the capital city of New Zealand, increased views of blue space (both sea and freshwater) are associated with decreased psychological stresses [31]. Photos containing rivers, lakes, and coasts tend to be a stronger visual stimuli regarding perceived restoration than those without aquatic elements [32]. Listening to water-based sounds (e.g., river, stream, ocean waves, or waterfalls) can also reduce experimentally induced stresses faster than urban sounds, silence, and calming music [33].

Research relies on place-based or people-based indicators for the availability of green/blue spaces around people [34]. To measure the quantity of green/blue space, a large number of studies use indices generated from satellite-based images (e.g., the Normalized Difference Vegetation Index), and green/blue space volume, calculated using surveyed land-use maps at various scales (e.g., ground level visibility, buffer from home, community, neighborhood, and county borders) [1,18,19,20,31,35,36]. Another popular measurement is proximity to green/blue space by closest linear distance or road network-based distance [18,37]. Because place-based indicators only reflect green/blue space availability objectively [37], researchers have begun to capture how people actually visit them, through observing on-site usability, interviewing people’s usage habits, tracing visitors’ movement with GPS or relying on highly aggregated geolocation data (e.g., cellphone or Twitter locations) [24,38,39,40,41,42]. These approaches have been criticized due to their high labor investment and biased sample selection (e.g., limited access to smartphone or digital equipment among older and younger populations) [41].

As more than half of world’s population lives in urban areas, the negative impacts of urbanization, including environmental pollution, urban heat island effect, etc., are threatening increasing numbers of people [43]. This is particularly challenging for the older population, which is often more vulnerable to environmental deterioration [18,19]. Given the potential benefits of green/blue spaces, municipalities across the global have invested significant efforts into naturalizing communities with green and blue spaces [44]. Positive outcomes of these efforts on senior people’s health have been largely confirmed in developed countries but similar observation is limited in rapidly urbanizing low- and middle-income Asian countries [19].

Accommodating the largest number of older people in the world, China is experiencing rapid population aging. In 2010, the number of senior people above the age of 60 was more than 177.6 million, accounting for 13.26% of the total population [45]. Moreover, the proportion of senior people is projected to rise to around 25% by 2030 [46]. Meanwhile, growing urbanization and industrialization pose considerable threats to local environments across the country, to which people of advanced ages are particularly sensitive [18,19]. The government has invested in green and blue spaces as one of the major tools to fight against environmental deterioration. Significant efforts have been put into practice in the past two decades to create green and blue spaces around neighborhoods by planting more trees, eliminating water pollution, and restoring concrete rivers and lakes [47]. However, the health benefits of such efforts have received less attention at the national level, particularly regarding the effects of blue spaces on the elderly. Therefore, the topic deserves scrutiny to support policymakers and urban planners.

This study examines how the availability of green and blue spaces is associated with general health among senior populations in China. To our best knowledge, this is the first attempt to examine green and blue spaces at the neighborhood level across China. Relying on a large-scale national survey, we examined how the self-rated health (SRH) of senior individuals is associated with the following factors: (1) green and blue space coverage within 1 km from a neighborhood, (2) closest distance to parks (only in urban areas), and (3) closest distance to different types of waterbodies.

## 2. Materials and Methods

### 2.1. Outcome Variable: Self-Rated Health

This study relies on a recently released national-scale social survey, the Chinese Social Survey (CSS). Launched by the Chinese Academy of Social Science in 2005, the CSS has been conducted every two years, relying on a multistage, probability-proportional-to-size sampling approach across the entire country [48]. Each selected participant above age 18 was interviewed in person by trained interviewers under the supervision of investigators [48].

We used the 2011 wave as this was the only cohort that recorded the SRH of senior populations (age above 60 as a threshold). A one-item measure for SRH was asked for each participant above age 60: “How do you rate your current health conditions in general?” The answers are coded on a 4-point scale as “3” (good health), “2” (fair health), “1” (poor health), and “0” (cannot take care of myself). The category “0” (cannot take care of myself) was eliminated as these responses came from an advanced aging population (above 80), who confront a higher risk of mortality and rarely visit or contact green or blue spaces daily [18]. The total number of observations in the 2011 CSS was 7036, which were collected from 148 cities in 30 provinces in China. Of that total, 1773 observations were of senior populations and included in our analysis. Observations with null values were excluded when respondents were allowed not to answer individual questions.

Another important feature of the CSS 2011 wave was that it is the only version recording detailed local data such as the address of each respondent (on a scale similar to the neighborhood level in the United States). Two trained research assistants manually searched each geolocation through Gaode Map (AutoNavi Software Co., Ltd., Beijing, China) (similar to Google Maps, with a geocoding function). The authors also double-checked all the returned geocodes on Gaode Map to ensure accuracy. Thus, the nearby availability of green and blue spaces and other location-specific environmental factors could be calculated, and the relationship between SRH and green or blue space availability could be analyzed.

### 2.2. Predictor Variables: Availability of Green and Blue Space

To measure the availability of green space, two indicators were used: overall green space coverage ratio and the closest linear distance to a park (in an urban area). Following a study at the same neighborhood scale, a green space coverage ratio was measured within a 1 km buffer from the centroid of neighborhoods as a proxy for total vegetation exposure around respondents’ homes [18]. The green space coverage ratio can reflect accumulated opportunities to visit and contact green spaces around homes, and thus gaining multiple health benefits [35]. It was calculated based on the following steps: vegetation pixels were extracted from preprocessed Landsat images using an NDVI (normalized difference vegetation index) value of 0.1 as a threshold, according to a study using the same dataset [49]. Next, the coverage ratio was generated from the total area of extracted vegetation pixels divided by the area of 1 km buffer from the neighborhood centroid. The Landsat images were derived from Global Forest Change Data with a resolution of 30 m [50]. The dataset provided composite imagery based on median observations from a set of quality assessed, cloud-free, growing season observations in four spectral bands, namely, Landsat bands 3 (RED), 4 (NIR), 5 (SWIR), and 7 (SWIR) [50]. The quality assessment included image resampling, conversion of raw digital numbers to top-of-atmosphere reflectance, cloud/shadow/water screening, and image normalization [50]. Thus, our study conducted no further atmospheric corrections. Despite scholars suggesting that higher resolution satellite images (e.g., Sentinel) can better reflect the green space volume [1], these datasets are subject to limited temporal and spatial availability. For example, Sentinel data (the most recent images were taken in around 2016 in China) did not cover the time and location in which the survey was taken (year 2011). Instead, the Landsat images provide better availability and consistent coverage and thus were used in the analysis. A study has also confirmed that NDVI calculated in Landsat images and high-resolution land use satellite images (3ft) were highly correlated (r = 0.87–0.97) [51].

Additionally, we calculated the closest linear distance to the entrance to a park, as this provides major accessible vegetated spaces for the public’s outdoor activities in urban areas. The park locations were also extracted from Gaode Map, which records the entrances in the form of “Points of Interest.” The closest distance to a park was only entered into regression analyses for urban areas as rural areas had no parks. It is admitted that the distances measured by a network-based approach can be more actual than a linear distance. However, the results calculated by the two types of measurement are often highly correlated [52,53,54], and their associations with health outcomes also present consistent trends [55]. The second reason we used a linear distance is that the green space planning sector in China adopts it to measure the service capacity of parks, and one of our goals was to test the effectiveness of such measurements for more direct planning references.

Regarding blue space, we measured the coverage ratio of all types of water surfaces and the closest linear distance to different types of blue spaces. The data on water surfaces were derived from the Time Series of Inland Surface Water Dataset in China (ISWDC), which mapped inland waterbodies larger than 0.0625 km^2^ in the terrestrial land of China for the period 2000–2016 in an 8-day temporal and 250-m spatial resolution [56]. We aggregated maps in 2011 to include all water surfaces in our analysis. The coverage ratio of water surfaces was calculated again with a 1-km buffer from the centroid of respondents’ neighborhoods. Considering the different health effects of the different types of waterbodies identified in the abovementioned studies [2,18,19,27,57], we differentiated three types of blue space, namely rivers, lakes, and coasts, and measured the closest distance from neighborhood centroids to each type.

### 2.3. Covariates: Individual and Neighborhood-Level Attributes

**Figure 1:**
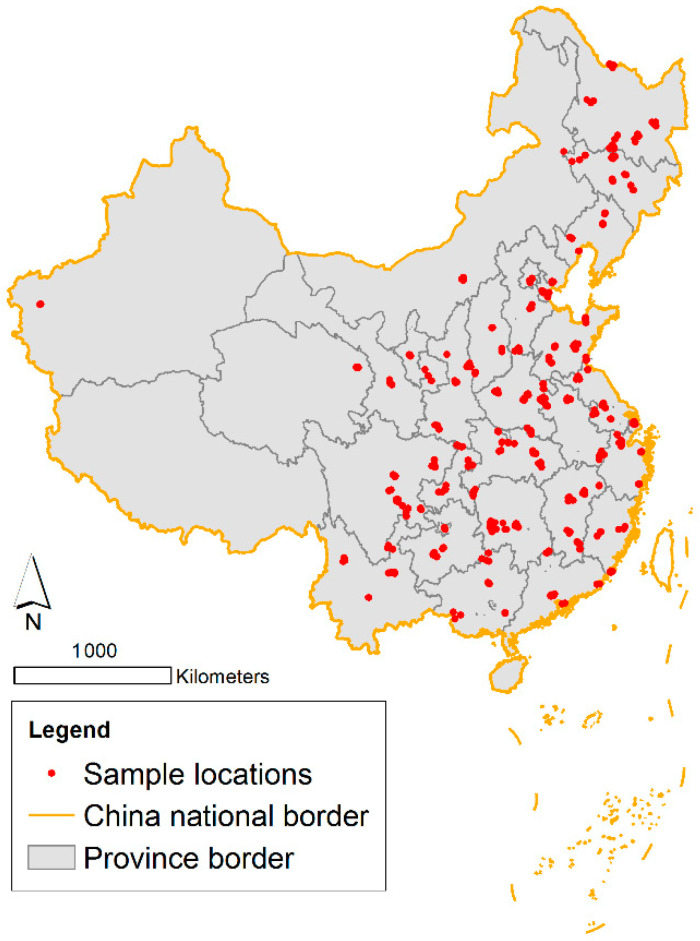
Study area and subdistrict locations of the sample.

Individual-level socioeconomic characteristics were collected in CSS 2011 regarding the respondents’ age, marital status, ethnicity, insurance, lifestyle, education, household registration location (hukou in Chinese), occupation, income, and assets [48]. The survey also documented the types of communities, housing statuses, and urban-rural codes according to official documentation [48,58]. Neighborhood-level features include distance to a major road, population density, and GDP production per km^2^ as control variables. The major road categories include national roads, provincial roads, and ordinary roads that are similar to the US highway [59]. Table 1 demonstrates all data sources involved in this study. Sample locations are mapped in Figure 1.

### 2.4. Statistical Analysis

To identify the association between senior people’s SRH and the availability of green and blue spaces, we used multiple multivariate regression in the form of ordinary least squares (OLS) regression. The OLS model is easier to interpret than the ordered probit model [67,68,69]. In equation (1), we regressed the SRH (Y) of each respondent (i) as the dependent variable on green ($Green_{i}$) and blue ($Blue_{i}$) space-related indicators—coverage ratio of green/blue space areas and closest distance to different types of green/blue spaces—as independent variables while adjusting for other individual covariates (IND). Housing type (HOUSE), community type (COMM), and city level (CITY) fixed effects were also entered as dummy variables in the models. $\varepsilon_{i}$ is an error term. Statistical descriptions of each variables are shown in Table 2.

(1) $$Y_{i}=\;\beta_{0}+\beta_{1}Green_{i}+\;\beta_{2}Blue_{i}+IND+HOUSE+\;COMM+CITY+\varepsilon_{i}$$

The regression was conducted separately on urban and rural areas, given widely varying geographical and socioeconomic features. Sensitivity tests were performed by (1) using different buffer distances (0.5 km, 2 km, and 3 km) to calculate the green and blue space coverage ratio, and (2) categorizing continuous variables (distance to green and blue spaces) into dummies. The distances to parks, roads, and rivers were entered in logarithmic forms due to the detection of nonlinearity. Distances to lake and coast were excluded due to identified collinearities. We used distances of 300 m and 500 m as thresholds for creating dummies. A 300 m distance reflects a 5-min walking time for older people with or without mobility issues [70]. A 500 m distance is used officially in urban planning evaluations for parks in China [71]. Distances to shorelines and lakes used 1 km or 5 km as a threshold because there are no samples within a shorter distance, such as 1 km from lakes. Five km from a major lake or coast could be used as a valid threshold, as a study found that people living within 5 km often presented higher mental and general health [24]. A variance inflation factor (VIF) was tested, which returned a maximal value of 8.7 across all models. This finding indicates little collinearity present in our models (using 10 as a suggested threshold). All of the statistical analyses were performed in Stata 15 (StataCorp LLC., College Station, TX, USA).

## 3. Results

### 3.1. Descriptive Statistics

The average SRH among senior populations above age 60 in China was 1.967 (S.D. = 0.706), with residents from urban areas having a slightly better value of 2.011 (S.D. = 0.685) than that reported by rural residents of 1.901 (S.D. = 0.733) (Table 2). The higher the SRH value, the healthier a respondent rated. 24.4% of people in urban areas reported “good health” status, while the number was 22.5% in rural areas. Regarding the availability of green and blue spaces, the average coverage ratio of waterbodies within 1 km from the centroid of a neighborhood was 0.035 (S.D. = 0.072) in urban areas and was a low 0.010 (S.D. = 0.048) in rural areas. The average green space coverage ratio was 0.339 (S.D. = 0.273) in urban locations and was much higher, 0.830 (S.D. = 0.245), in rural areas. In urban areas, the average distance to the closest park was 3167 m (S.D. = 7144 m, ranging from 19 m–45,649 m). The mean distance to the closest waterbody was 2944 m (S.D. = 4595 m, ranging from 1 m–48,240 m) in urban areas, and 8135 m (S.D. = 8652 m, ranging from 1 m–44,417 m) in rural areas. The percent of urban residents living within 1 km of a waterbody larger than 6.25 ha was 39.8%, and only 8.4% of rural residents were living within the same distance to a waterbody.

### 3.2. Multivariate Regressions

Table 3 reports the influences of green and blue spaces around neighborhoods on older persons’ SRH while controlling for individual and other environmental attributes in urban and rural areas. In Model 1 and Model 4, the coverage ratios of green and blue spaces returned insignificant results, suggesting that the quantity of overall green and blue space did not exert any impact on SRH. The sensitivity test results showed similar trends at different coverage distances as can be seen in Table A2 in Appendix A. In Model 2, the nearest distance to a park was negatively associated with SRH but was statistically insignificant. The nearest distance to a waterbody also presented insignificant impacts on SRH in both urban and rural areas (Model 2 & 5). However, senior people living within 0.3 km of a waterbody reported better SRH (*p* value < 0.05). Having a major river (within 0.3–0.5 km) or coastline (within 1 km and 1–5 km) in the vicinity of an urban respondent’s home might negatively affect SRH (*p* value < 0.1), though such a trend was marginal in rural areas. Living near a coastline posed higher negative impacts on SRH of senior people than living close to a major river.

Detailed regression results are shown in Table A1 in Appendix A. As expected, in urban areas, advancing age had negative influences on people’s SRH conditions. Males tended to show better SRH than female seniors, and married people reported a better health condition than those unmarried. However, all three trends were minimal in rural locations. Being employed, retired, or a full-time homemaker could benefit a senior’s health status, but only in urban areas. Higher education levels also contributed positive effects to SRH in both urban and rural areas. Older people living in economically developed regions, as proxied by GDP per km^2^, tended to have higher SRH.

## 4. Discussion

Using multiple indicators to measure the availability of green and blue space, this study examined how these were related to older people’s SRH status across China. Spatial-explicit data on green and blue spaces as well as other local environmental factors were obtained based on a nationwide social survey with detailed geolocation of respondents’ neighborhoods. Urban and rural areas were separately entered into regression estimations. Our results did not find strong evidence that living with a higher amount of green/blue spaces or within closer distance to green/blue spaces contributes to higher SRH among senior populations. A clear urban-rural difference was observed in that blue space tends to pose negative impacts in SRH in urban areas as nonsignificant outcomes were found in rural areas. Living close to certain types of waterbodies, such as rivers and coastlines, might be detrimental to older people’s health in urban areas. By offering a national observation in a developing region, this study enriches the current literature on public health and the environment, with a particular focus on green and blue spaces. The results provide reference information to assist decision-makers and urban planners to manage urbanization in an aging era.

### 4.1. Associations between Green Space and Seniors’ SRH

Our study identified both coverage of and distance to green space pose insignificant impacts on senior’s SRH. Despite the widely acknowledged positive impacts of green space on people’s general health, many studies returned mixed outcomes when investigating subpopulations, such as older people [72]. For example, a study of 260,061 Australians over 45 years old identified that people living in the greenest neighborhood had a lower risk of psychological distress [73]. Similarly, a nationwide study in the Netherlands in both urban and rural areas confirmed that self-perceived health of both younger and older groups were higher in areas with more green spaces [74]. A city-level study in Wuhan, China also found that older people with greater access to parks had a lower chance of cardio-cerebral vascular diseases, joint diseases and endocrine disease [75]. On the contrary, a nation-level health survey in Canada using 69,910 individuals who were 20 years of age and older revealed an insignificant association between physical activity and green space quantity for older people (age above 60), which demonstrated less health benefits older people might obtain from green spaces [17]. A health study in Berlin, Germany, did not find strong associations between self-reported health of older people (age above 50) and the closest distance to urban green spaces [76]. Using the same neighborhood-level green space coverage, Liu, et al. [77] found that the coverage ratio is insignificant after controlling for time spent on physical activity, frequency of stressful experiences, and neighborhood social cohesion.

The insignificant impacts of green space on people’s health might be due to the characteristics of the older population in our study, who are often less inclined to travel longer distances to use green spaces due to physical limitations and long-term illnesses [17,18], and thus cannot actively use green spaces to promote their health. Another potential explanation is that green space coverage cannot capture different features or facilities such as desirable amenities and this affects the usability and preferences of older people as well as health benefits they might obtain [3,78]. Moreover, varying outcomes of vegetation coverage on SRH might relate to the measurement approaches concerning the selection of areal units such as geographical borders of census units, buffer distances from home entrances, or neighborhood and subdistrict centroids [36,79]. Using green space coverage within a 250-m buffer from exact residential addresses, a research in Berlin found that the coverage ratio positively impacts people’s SRH status [76]. In contrast, a study in the United States found that the overall green space volume within a zip code border is not associated with people’s general health status in both urban and rural areas [80]. After adjusting for confounders, the presence of larger-scale, city-level greenness is also confirmed to have insignificant impacts on several diseases, such as heart problems, diabetes, and lung cancer in the United States [81].

### 4.2. Associations between Blue Space and Seniors’ SRH

The coverage of water surfaces around neighborhoods as well as close distances to different types of waterbodies presents few positive impacts on older people’s SRH in both urban and rural areas. This result contrasts with previous observations in developed regions as studies showed in Canada [39], England [24], Australia [28], Germany [82], New Zealand [31], and Spain [25], that living or being visually close to water surfaces can promote both mental and general health [2]. Another systematic literature review of 35 studies worldwide also confirmed in 22 of the studies that positive associations were shown between exposure to blue spaces and people’s well-being [83]. However, two studies on individual cities in China reported similar observations to our study that the water-coverage ratio was insignificant for older people’s well-being. The percentage of surrounding blue spaces within a 1-km buffer from a neighborhood had no impact on older people (above age 60) in Shanghai [18]. Additionally, the ratio of water in a 1-km buffer zone of the neighborhood’s boundary in Guangzhou also showed little relation to elderly individuals’ mental health, stress, physical activity, and social contacts [19].

One potential speculation on the minimal positive health effect of blue space is that the water-coverage ratio can hardly capture actual pathways showing how older people benefit from it. The actual quality and features of blue spaces might more substantially affect older people′s actual usages. Scholars have argued that senior populations are more concerned about safety (e.g., crime, biking), accessibility (e.g., heavy traffic), or other public facilities (e.g., restrooms, seating facilities), the lack of which might discount the attractiveness of a waterbody for them, and thus discourage their usage for health promoting activities [2,39,84]. As with green spaces, the older people’s physical constraints might also inhibit their frequent visitations to blue spaces, which further mitigate the health benefits they might obtain from actively engaging with waterbodies through walking or visual/acoustical contacts [2,3,19]. Additionally, the coverage ratio measures water availability from above based on satellite images and hardly reflects ground-level exposures, such as visibility of the waterbody, which has been proven to reduce depressive symptoms among older people above 60 [20] and is particularly important for maintaining the wellbeing of seniors [85]. Another explanation for the results of this study might be the small inter-neighborhood variance in the waterbody ratio [18]. That is, in both urban and rural areas, more than 90% of observations have water coverage that is below 8% on average.

The closest distances to various types of waterbody were not associated with the elderly’s SRH. Worse, living close to rivers or coastal areas negatively affected the reported general health statuses. Such negative impacts could be due to the poor or polluted water quality in rivers or coastlines, which might be prevalent in developing countries such as China [2]. For example, in urban areas of Guangzhou, China, the river quality was much lower than in rural regions, according to the Guangzhou Municipal Bureau of Ecological Environment. Additionally, the lower mobility of older populations tends to expose persons longer to polluted water if they visit or stroll around a river, subsequently increasing the likelihood of receiving negative or even detrimental effects [19].

### 4.3. Limitations and Future Studies

Our study confronts several potential limitations regarding data and scale. Despite employing fine-resolution neighborhood locations for measuring green and blue spaces, the measurement might be less accurate than using exact residential addresses. A neighborhood often contains several residential quarters in China. The size of the neighborhood in urban areas is smaller than that in rural areas. Thus, urban areas might capture surrounding environmental characteristics such as green and blue coverage more accurately. Types of green and blue spaces might still be too coarse to reflect detailed features or qualities. Rivers, lakes, small waterbodies, and parks might present different qualities and features. For instance, other types of green spaces, such as pocket gardens or informal green spaces, might provide recreational services such as large parks do [86]. Heavily polluted waterbodies along a river or higher pollen exposure in a vegetation-rich park might directly harm people’s health, particularly sensitive senior populations, by causing allergic reactions or respiratory disorders [19,76]. Including detailed river or park data such as water quality, waterfront accessibility, maintenance, travel barriers, facilities, etc. might assist to further explain the health effects of green/blue spaces. Outcomes are also related to how people perceive and actually use green and blue spaces [3,39,87]. The indicators involved in this study, such as overall coverage ratio and closest distance, reflect the objective availability of green and blue spaces, which does not unravel potential pathways of how people actually benefit from visiting and contacting with these spaces. For example, if the closest park lacks proper amenities or is unsafe, older people might be less willing to visit and subsequently could not gain benefits from the park. These limitations suggest future studies that include the exact addresses of surveys, more detailed features of green and blue spaces, as well as perceptions and actual usages, particularly among senior populations, to reduce potential bias.

## 5. Conclusions

Relying on a recently released national-scale survey (the China Social Survey, 2011 wave), this study examined fine-grained, neighborhood-level availability of green and blue spaces and the associations with the general health of senior people across China. We found that the coverage ratio of overall green spaces and waterbodies around a resident’s home had marginal effects on the SRH status of seniors in both urban and rural areas. Regarding different types of green and blue spaces, the presence of a major river (within 0.3–0.5 km) or coastline (within 1 km and 1–5 km) in the vicinity of home negatively affects SRH among the elderly in urban areas. Proximity to lakes or parks did not exert significant influences on SRH in older people. In urban area, older people living within 0.3 km from any types of waterbodies with surface measurements greater than 6.25 ha reported better SRH. As a pioneering study on nationwide public health effects of green and blue spaces, the empirical evidence expands current discourses on health outcomes influenced by local environmental features. In particular, this work adds to the less-explored literature on the impact of blue spaces on health particularly among senior populations, adding a national-level investigation from a developing country.

## Figures and Tables

**Table 1:** Summary of datasets used in this study.

Name of Data	Sources	Year of Data Collected
Chinese Social Survey (CSS)	Li, Li, Chen, Zou, Cui, Ren, Tian, Zhang, Fan, Wang and Hu [48]	2011
Neighborhood location (point)	Gaode Map [60]	2010
Landsat images (30 m)	Hansen, Potapov, Moore, Hancher, Turubanova, Tyukavina, Thau, Stehman, Goetz, Loveland, Kommareddy, Egorov, Chini, Justice and Townshend [50]	2012
Park entrance (point)	Gaode Map [60], Google [61]	2010
Water surface area (250 m)	Lu, Ma, Ma, Tang, Zhao and Hasan Ali Baig [56]	2011
China major lake (polygon)	National Geomatics Center of China [62]	2014
China river (polyline)	National Geomatics Center of China [63]	2011
Coastal line (polyline)	Wessel and Smith [64]	2010
China population density (1 km)	Xu [65]	2010
China GDP per area (1 km)	Xu [66]	2010
China major road	State Bureau of Surveying and Mapping of China [59]	2009

**Table 2:** Descriptive statistics of all variables.

	Urban (*n* = 1061)				Rural (*n* = 712)			
Variables	Mean	S.D.	Min.	Max.	Mean	S.D.	Min.	Max.
**Outcome**								
Self-rate health	2.011	0.685	1.000	3.000	1.901	0.733	1.000	3.000
**Predictors**								
Coverage of waterbody in 1 km	0.035	0.072	0.000	0.380	0.010	0.048	0.000	0.460
Coverage of vegetation in 1 km	0.339	0.273	0.000	1.000	0.830	0.245	0.064	0.999
The nearest distance to park (m)	3167	7144	19	45,649				
The nearest distance to waterbody (m)	2944	4595	1	48,240	8135	8652	1	44,417
Nearest distance to river (m)	4497	6126	28	29,731	9138	7548	25	38,546
Within 0–0.3 km of park	0.237	0.425	0.000	1.000				
Within 0.3–0.5 km of park	0.171	0.377	0.000	1.000				
Within 0.5–1 km of park	0.259	0.438	0.000	1.000				
Within 0–0.3 km of waterbody	0.134	0.341	0.000	1.000	0.018	0.134	0.000	1.000
Within 0.3–0.5 km of waterbody	0.069	0.254	0.000	1.000	0.041	0.197	0.000	1.000
Within 0.5–1 km of waterbody	0.194	0.395	0.000	1.000	0.025	0.157	0.000	1.000
Within 0–0.3 km of river	0.065	0.246	0.000	1.000	0.040	0.197	0.000	1.000
Within 0.3–0.5 km of river	0.033	0.180	0.000	1.000	0.014	0.118	0.000	1.000
Within 0.5–1 km of river	0.135	0.342	0.000	1.000	0.032	0.177	0.000	1.000
Within 1 km of coastline	0.001	0.030	0.000	1.000	0.000	0.000	0.000	0.000
Within 1–5 km of coastline	0.071	0.257	0.000	1.000	0.007	0.083	0.000	1.000
Within 5 km of lake	0.003	0.053	0.000	1.000	0.027	0.161	0.000	1.000
**Covariates**								
Demographical information								
Age	69.5	7.3	60	92	68.5	7.0	60	101
Gender (1 = male, 0 = female)	0.469	0.499	0.000	0.000	0.513	0.500	0.000	1.000
Ethnic group (1 = Han, 0 = other)	0.948	0.222	0.000	1.000	0.885	0.319	0.000	1.000
Marriage (1 = married, 0 = single)	0.994	0.080	0.000	1.000	0.985	0.123	0.000	1.000
Local Hukou (1 = local, 0 = migrant)	0.783	0.412	0.000	1.000	0.966	0.180	0.000	1.000
Number of person(s) in household	4	2	1	21	4	2	1	21
Living alone (1 = yes, 0 = no)	0.631	0.483	0.000	1.000	0.624	0.485	0.000	1.000
Occupation (1 = yes, 0 = no)								
Employed	0.149	0.356	0.000	1.000	0.628	0.484	0.000	1.000
Not able to work	0.122	0.328	0.000	1.000	0.273	0.446	0.000	1.000
Retired	0.604	0.489	0.000	1.000	0.052	0.222	0.000	1.000
Homemaker	0.056	0.229	0.000	1.000	0.020	0.139	0.000	1.000
Income level (1 = yes, 0 = no)								
<5000 CNY	0.156	0.363	0.000	1.000	0.552	0.498	0.000	1.000
5000–15,000 CNY	0.181	0.385	0.000	1.000	0.232	0.423	0.000	1.000
15,000–30,000 CNY	0.407	0.492	0.000	1.000	0.091	0.288	0.000	1.000
>30,000 CNY	0.156	0.363	0.000	1.000	0.017	0.129	0.000	1.000
No answer	0.100	0.300	0.000	1.000	0.108	0.310	0.000	1.000
Education level (1 = yes, 0 = no)								
Below elementary school (lowest)	0.189	0.392	0.000	1.000	0.407	0.492	0.000	1.000
Elementary school	0.287	0.453	0.000	1.000	0.434	0.496	0.000	1.000
Middle school	0.240	0.427	0.000	1.000	0.130	0.337	0.000	1.000
High school	0.083	0.277	0.000	1.000	0.007	0.083	0.000	1.000
Technical secondary school	0.083	0.277	0.000	1.000	0.014	0.118	0.000	1.000
Technical Junior college	0.005	0.068	0.000	1.000	0.003	0.053	0.000	1.000
Junior college	0.061	0.240	0.000	1.000	0.004	0.065	0.000	1.000
College	0.051	0.220	0.000	1.000	0.001	0.037	0.000	1.000
Graduate (highest)	0.001	0.030	0.000	1.000	0.000	0.000	0.000	0.000
**Assets**								
Pension benefit (1 = yes, 0 = no)	0.681	0.466	0.000	1.000	0.376	0.485	0.000	1.000
Medical insurance (1 = yes, 0 = no)	0.881	0.324	0.000	1.000	0.910	0.286	0.000	1.000
Owns car (1 = yes, 0 = no)	0.074	0.262	0.000	1.000	0.035	0.184	0.000	1.000
Owns housing property	1.062	0.549	0.000	5.000	1.136	0.467	0.000	6.000
**Environmental features**								
The nearest distance to road (m)	1843.3	2237.9	7.0	22,037.6	5365.6	4831.9	7.7	23,496.0
GDP per km^2^ (10,000 CNY)	11,660.4	10,720.8	0.0	50,051.8	1844.5	4081.1	0.0	26,769.4
Number of person(s) per km^2^	10,707.6	10,187.3	0.0	41,878.0	638.2	1164.4	0.0	14,839.0

**Table 3:** Regression results (shortened).

	Urban (*n* = 1061)			Rural (*n* = 712)		
Variables	Model 1	Model 2	Model 3	Model 4	Model 5	Model 6
Coverage of waterbody in 1 km	−0.498			0.212		
	(0.474)			(0.716)		
Coverage of vegetation in 1 km	−0.200			−0.048		
	(0.176)			(0.316)		
The nearest distance to park (log)		−0.031				
		(0.027)				
The nearest distance to waterbody (log)		−0.029			0.028	
		(0.028)			(0.045)	
The nearest distance to river (log)		0.038			−0.008	
		(0.036)			(0.035)	
Within 0–0.3 km of park			0.033			
			(0.091)			
Within 0.3–0.5 km of park			−0.056			
			(0.097)			
Within 0.5–1 km of park			0.033			
			(0.088)			
Within 0–0.3 km of waterbody			0.210 *			−0.013
			(0.100)			(0.319)
Within 0.3–0.5 km of waterbody			0.124			−0.437
			(0.115)			(0.290)
Within 0.5–1 km of waterbody			0.041			0.194
			(0.070)			(0.195)
Within 0–0.3 km of river			−0.099			0.195
			(0.144)			(0.217)
Within 0.3–0.5 km of river			−0.237 ^+^			0.388
			(0.140)			(0.430)
Within 0.5–1 km of river			−0.093			0.202
			(0.085)			(0.252)
Within 1 km of coastline			−1.196 ^+^			
			(0.686)			
Within 1–5 km of coastline			−0.379 ^+^			−0.969
			(0.200)			(0.629)
Within 5 km of lake			−0.156			0.091
			(0.515)			(0.235)
						
Constant	1.810 ***	1.807 ***	1.758 ***	2.278 **	2.065 *	2.308 **
	(0.464)	(0.544)	(0.473)	(0.763)	(0.846)	(0.723)
Observations	1061	1061	1061	712	712	712
R-squared	0.215	0.214	0.225	0.230	0.232	0.241
Adjusted R-squared	0.109	0.104	0.110	0.095	0.097	0.097
F test model	2.03	1.95	1.96	1.71	1.70	1.68
*p*-value of F model	0.000	0.000	0.000	0.000	0.000	0.000

Standard errors in parentheses; *** *p* < 0.001, ** *p* < 0.01, * *p* < 0.05, ^+^ *p* < 0.1; Socioeconomic, housing, community, and city related control variables were included by not reported; See Table A1 in Appendix A for full regression results.

## Data Availability

All the data used in this study was publicly available and the sources and described in text in detail.

## Appendix A

**Table A1:** Detailed regression results.

	Urban (*n* = 1061)			Rural (*n* = 712)		
Variables	Model 1	Model 2	Model 3	Model 4	Model 5	Model 6
Coverage of waterbody in 1 km	−0.498			0.212		
	(0.474)			(0.716)		
Coverage of vegetation in 1 km	−0.200			−0.048		
	(0.176)			(0.316)		
The nearest distance to park (log)		−0.031				
		(0.027)				
The nearest distance to waterbody (log)		−0.029			0.028	
		(0.028)			(0.045)	
The nearest distance to river (log)		0.038			−0.008	
		(0.036)			(0.035)	
Within 0–0.3 km of park			0.033			
			(0.091)			
Within 0.3–0.5 km of park			−0.056			
			(0.097)			
Within 0.5–1 km of park			0.033			
			(0.088)			
Within 0–0.3 km of waterbody			0.210 *			−0.013
			(0.100)			(0.319)
Within 0.3–0.5 km of waterbody			0.124			−0.437
			(0.115)			(0.290)
Within 0.5–1 km of waterbody			0.041			0.194
			(0.070)			(0.195)
Within 0–0.3 km of river			−0.099			0.195
			(0.144)			(0.217)
Within 0.3–0.5 km of river			−0.237 ^+^			0.388
			(0.140)			(0.430)
Within 0.5–1 km of river			−0.093			0.202
			(0.085)			(0.252)
Within 1 km of coastline			−1.196 ^+^			
			(0.686)			
Within 1–5 km of coastline			−0.379 ^+^			−0.969
			(0.200)			(0.629)
Within 5 km of lake			−0.156			0.091
			(0.515)			(0.235)
Age	−0.014 ***	−0.014 ***	−0.013 ***	0.003	0.004	0.003
	(0.003)	(0.003)	(0.003)	(0.005)	(0.005)	(0.005)
Gender	0.145 **	0.139 **	0.157 ***	0.003	−0.005	0.004
	(0.046)	(0.047)	(0.046)	(0.065)	(0.065)	(0.065)
Ethnic group	0.099	0.096	0.093	0.059	0.072	0.058
	(0.112)	(0.113)	(0.113)	(0.141)	(0.144)	(0.141)
Marriage	0.515 ^+^	0.509 ^+^	0.533 *	−0.301	−0.314	−0.311
	(0.262)	(0.266)	(0.264)	(0.232)	(0.233)	(0.232)
Local Hukou	−0.061	−0.057	−0.063	−0.231	−0.247	−0.259
	(0.056)	(0.057)	(0.056)	(0.167)	(0.170)	(0.168)
No. of person(s) in household	0.003	0.001	0.003	0.009	0.010	0.012
	(0.012)	(0.012)	(0.012)	(0.016)	(0.016)	(0.016)
Living alone	−0.018	−0.034	−0.022	−0.061	−0.061	−0.057
	(0.048)	(0.049)	(0.048)	(0.068)	(0.068)	(0.068)
Employed	0.373 ***	0.350 ***	0.371 ***	0.055	0.066	0.035
	(0.100)	(0.102)	(0.101)	(0.184)	(0.185)	(0.185)
Not able to work	0.080	0.062	0.074	−0.293	−0.286	−0.310
	(0.104)	(0.106)	(0.104)	(0.189)	(0.190)	(0.191)
Retired	0.209 *	0.201 *	0.196 *	−0.113	−0.099	−0.176
	(0.094)	(0.095)	(0.094)	(0.236)	(0.237)	(0.239)
Homemaker	0.430 ***	0.415 **	0.411 **	0.243	0.254	0.234
	(0.125)	(0.126)	(0.125)	(0.272)	(0.273)	(0.274)
<5000 CNY as reference						
5000–15,000 CNY	0.049	0.047	0.054	0.087	0.088	0.083
	(0.081)	(0.082)	(0.082)	(0.074)	(0.074)	(0.074)
15,000–30,000 CNY	−0.027	−0.037	−0.034	0.109	0.110	0.123
	(0.088)	(0.089)	(0.089)	(0.120)	(0.120)	(0.120)
>30,000 CNY	0.074	0.070	0.067	−0.367	−0.376	−0.372
	(0.102)	(0.104)	(0.103)	(0.249)	(0.251)	(0.251)
No answer	0.113	0.113	0.121	−0.155	−0.156	−0.172
	(0.089)	(0.090)	(0.090)	(0.105)	(0.106)	(0.106)
Below elementary school as reference						
Elementary school	−0.114 ^+^	−0.101	−0.108	0.040	0.038	0.032
	(0.066)	(0.068)	(0.066)	(0.071)	(0.072)	(0.071)
Middle school	−0.011	−0.003	−0.001	0.076	0.082	0.080
	(0.077)	(0.078)	(0.077)	(0.101)	(0.102)	(0.101)
High school	0.075	0.100	0.092	0.307	0.318	0.318
	(0.100)	(0.101)	(0.100)	(0.364)	(0.364)	(0.362)
Technical secondary school	−0.068	−0.050	−0.058	0.602 *	0.669 *	0.630 *
	(0.098)	(0.100)	(0.099)	(0.275)	(0.289)	(0.277)
Technical Junior college	−0.314	−0.298	−0.327	−0.833	−0.862	−0.883
	(0.305)	(0.307)	(0.305)	(0.548)	(0.546)	(0.545)
Junior college	−0.041	−0.034	−0.032	0.490	0.475	0.538
	(0.112)	(0.115)	(0.113)	(0.463)	(0.465)	(0.463)
College	−0.076	−0.066	−0.084	0.798	0.829	0.924
	(0.121)	(0.122)	(0.121)	(0.743)	(0.751)	(0.756)
Graduate	0.098	0.063	0.046			
	(0.662)	(0.667)	(0.670)			
Pension benefit	0.071	0.076	0.083	−0.075	−0.071	−0.073
	(0.060)	(0.061)	(0.061)	(0.083)	(0.084)	(0.085)
Medical insurance	0.065	0.069	0.062	0.126	0.121	0.118
	(0.071)	(0.072)	(0.071)	(0.106)	(0.108)	(0.107)
Owns car	0.074	0.073	0.063	0.175	0.189	0.128
	(0.086)	(0.087)	(0.086)	(0.160)	(0.164)	(0.162)
Owns housing property	0.037	0.037	0.034	0.071	0.069	0.070
	(0.041)	(0.042)	(0.041)	(0.067)	(0.068)	(0.068)
The nearest distance to road (log)	0.030	0.033	0.019	−0.016	−0.017	−0.015
	(0.023)	(0.025)	(0.024)	(0.032)	(0.032)	(0.032)
GDP per km^2^	0.001 *	0.001 ^+^	0.001 *	0.001	0.001	−0.001
	(0.000)	(0.000)	(0.000)	(0.000)	(0.000)	(0.000)
Number of person(s) per km^2^	0.000	0.000	0.000	−0.000	0.000	0.000
	(0.000)	(0.000)	(0.000)	(0.000)	(0.000)	(0.000)
Constant	1.810 ***	1.807 ***	1.758 ***	2.278 **	2.065 *	2.308 **
	(0.464)	(0.544)	(0.473)	(0.763)	(0.846)	(0.723)
Observations	1061	1061	1061	712	712	712
R-squared	0.215	0.214	0.225	0.230	0.232	0.241
Adjusted R-squared	0.109	0.104	0.110	0.095	0.097	0.097
F test model	2.03	1.95	1.96	1.71	1.70	1.68
*p*-value of F model	0.000	0.000	0.000	0.000	0.000	0.000
Community type dummy	Entered	Entered	Entered	Entered	Entered	Entered
Housing type dummy	Entered	Entered	Entered	Entered	Entered	Entered
City dummy	Entered	Entered	Entered	Entered	Entered	Entered

Standard errors in parentheses; *** *p* < 0.001, ** *p* < 0.01, * *p* < 0.05, ^+^ *p* < 0.1.

**Table A2:** Sensitivity test using different buffers for measuring green/blue coverage.

Variables	Urban Buffer 0.5 km	Urban Buffer 2 km	Urban Buffer 3 km	Rural Buffer 0.5 km	Rural Buffer 2 km	Rural Buffer 3 km
Coverage of waterbody in 0.5 km	−0.274			0.298		
	(0.453)			(0.489)		
Coverage of vegetation in 0.5 km	−0.172			0.050		
	(0.159)			(0.292)		
Coverage of waterbody in 2 km		0.103			0.176	
		(0.516)			(1.280)	
Coverage of vegetation in 2 km		−0.087			0.060	
		(0.207)			(0.384)	
Coverage of waterbody in 3 km			0.346			0.090
			(0.558)			(1.580)
Coverage of vegetation in 3 km			0.119			0.053
			(0.239)			(0.424)
Age	−0.014 ***	−0.014 ***	−0.014 ***	0.003	0.003	0.003
	(0.003)	(0.003)	(0.003)	(0.005)	(0.005)	(0.005)
Gender	0.145 **	0.144 **	0.143 **	0.001	0.003	0.003
	(0.046)	(0.046)	(0.046)	(0.065)	(0.065)	(0.065)
Ethnic group	0.097	0.096	0.100	0.060	0.060	0.060
	(0.112)	(0.113)	(0.113)	(0.141)	(0.141)	(0.142)
Marriage	0.515 ^+^	0.515 ^+^	0.508 ^+^	−0.303	−0.296	−0.296
	(0.263)	(0.263)	(0.263)	(0.232)	(0.232)	(0.232)
Local Hukou	−0.063	−0.066	−0.066	−0.230	−0.235	−0.234
	(0.055)	(0.056)	(0.055)	(0.167)	(0.167)	(0.167)
No. of person(s) in household	0.003	0.002	0.002	0.009	0.009	0.009
	(0.012)	(0.012)	(0.012)	(0.016)	(0.016)	(0.016)
Living alone	−0.021	−0.023	−0.025	−0.059	−0.062	−0.063
	(0.048)	(0.048)	(0.048)	(0.068)	(0.068)	(0.068)
Employed	0.370 ***	0.367 ***	0.362 ***	0.052	0.053	0.052
	(0.100)	(0.100)	(0.101)	(0.184)	(0.184)	(0.184)
Not able to work	0.075	0.075	0.067	−0.295	−0.292	−0.293
	(0.104)	(0.104)	(0.105)	(0.189)	(0.189)	(0.189)
Retired	0.209 *	0.207 *	0.203 *	−0.119	−0.116	−0.116
	(0.094)	(0.094)	(0.094)	(0.236)	(0.236)	(0.236)
Homemaker	0.423 ***	0.425 ***	0.419 ***	0.234	0.241	0.241
	(0.125)	(0.125)	(0.125)	(0.272)	(0.272)	(0.272)
<5000 CNY as reference						
5000–15,000 CNY	0.049	0.047	0.050	0.086	0.086	0.086
	(0.081)	(0.081)	(0.081)	(0.074)	(0.074)	(0.074)
15,000–30,000 CNY	−0.028	−0.031	−0.027	0.111	0.109	0.109
	(0.088)	(0.088)	(0.088)	(0.120)	(0.120)	(0.120)
>30,000 CNY	0.074	0.071	0.077	−0.366	−0.369	−0.367
	(0.102)	(0.102)	(0.103)	(0.249)	(0.249)	(0.250)
No answer	0.115	0.116	0.122	−0.155	−0.158	−0.158
	(0.089)	(0.089)	(0.089)	(0.105)	(0.105)	(0.105)
Below elementary school as reference						
Elementary school	−0.114 ^+^	−0.110 ^+^	−0.110 ^+^	0.042	0.040	0.040
	(0.066)	(0.066)	(0.066)	(0.071)	(0.071)	(0.071)
Middle school	−0.010	−0.005	−0.004	0.079	0.078	0.077
	(0.077)	(0.077)	(0.077)	(0.101)	(0.101)	(0.101)
High school	0.076	0.080	0.079	0.317	0.318	0.317
	(0.100)	(0.099)	(0.100)	(0.362)	(0.364)	(0.363)
Technical secondary school	−0.068	−0.062	−0.061	0.608 *	0.606 *	0.604 *
	(0.099)	(0.098)	(0.098)	(0.276)	(0.275)	(0.275)
Technical Junior college	−0.307	−0.304	−0.298	−0.849	−0.848	−0.847
	(0.305)	(0.305)	(0.305)	(0.547)	(0.546)	(0.546)
Junior college	−0.044	−0.040	−0.042	0.495	0.492	0.490
	(0.112)	(0.112)	(0.112)	(0.463)	(0.463)	(0.463)
College	−0.079	−0.077	−0.079	0.800	0.796	0.796
	(0.121)	(0.121)	(0.121)	(0.743)	(0.743)	(0.743)
Graduate	0.071	0.071	0.074			
	(0.662)	(0.663)	(0.663)			
Pension benefit	0.070	0.071	0.071	−0.074	−0.076	−0.075
	(0.060)	(0.060)	(0.060)	(0.083)	(0.084)	(0.084)
Medical insurance	0.065	0.072	0.071	0.127	0.125	0.125
	(0.071)	(0.071)	(0.071)	(0.106)	(0.106)	(0.106)
Owns car	0.075	0.076	0.077	0.172	0.175	0.174
	(0.086)	(0.086)	(0.086)	(0.160)	(0.160)	(0.160)
Owns housing property	0.036	0.038	0.039	0.070	0.072	0.072
	(0.041)	(0.041)	(0.041)	(0.067)	(0.068)	(0.068)
The nearest distance to road (log)	0.031	0.030	0.029	−0.016	−0.015	−0.015
	(0.024)	(0.023)	(0.023)	(0.032)	(0.032)	(0.032)
GDP per km^2^	0.001 *	0.001 *	0.001 *	0.001	0.001	0.001
	(0.000)	(0.000)	(0.000)	(0.000)	(0.000)	(0.000)
Number of person(s) per km^2^	0.000	0.000	0.000	−0.000	0.000	0.000
	(0.000)	(0.000)	(0.000)	(0.000)	(0.000)	(0.000)
Constant	1.787 ***	1.781 ***	1.747 ***	2.207 **	2.195 **	2.200 **
	(0.464)	(0.465)	(0.466)	(0.758)	(0.765)	(0.776)
Observations	1061	1061	1061	712	712	712
Adjusted R^2^	0.108	0.107	0.107	0.096	0.095	0.095
F test model	2.02	2.01	2.01	1.71	1.71	1.71
*p*-value of F model	0.000	0.000	0.000	0.000	0.000	0.000
Community type dummy	Entered	Entered	Entered	Entered	Entered	Entered
Housing type dummy	Entered	Entered	Entered	Entered	Entered	Entered
City dummy	Entered	Entered	Entered	Entered	Entered	Entered

Standard errors in parentheses; *** *p* < 0.001, ** *p* < 0.01, * *p* < 0.05, ^+^ *p* < 0.1.
